# Integrated single-cell and bulk transcriptome analysis reveal lactate metabolism-related signature and T cell alteration in atrial fibrillation

**DOI:** 10.3389/fcell.2025.1644702

**Published:** 2025-08-06

**Authors:** Xianglin Long, Junxi Li, Yeshen Zhang, Zhihui Zhang

**Affiliations:** ^1^ Department of Cardiology, The Third Xiangya Hospital of Central South University, Changsha, China; ^2^ Chongqing Medical University, Chongqing, China

**Keywords:** atrial fibrillation, lactate metabolism, t cell, transcriptomics, immunemetabolism

## Abstract

**Background:**

Atrial fibrillation (AF) is linked to modifications in T cell-mediated immunity. Although lactate metabolism influences T cell differentiation and function, its specific role in AF and associated immune processes remains inadequately understood.

**Methods:**

We performed an integrated transcriptomic analysis utilizing both bulk and single-nucleus RNA sequencing data derived from hearts exhibiting AF and those in sinus rhythm. Genes associated with lactate metabolism were curated from the MsigDB, and key genes were identified through Weighted Gene Co-expression Network Analysis and differential expression analysis. A diagnostic model based on machine learning was developed, and gene expression was further validated using qRT-PCR in a mouse model of AF. T cell heterogeneity was evaluated using the Seurat package, and intercellular communication was inferred using CellChat.

**Results:**

In AF, six key genes related to lactate metabolism showed transcriptomic changes linked to the AF phenotype and CD4+/CD8+ T cell populations. A diagnostic model using these genes achieved an AUC of 0.909 in external datasets. Single-nucleus RNA sequencing identified a reduced metabolism-related T cell subset (CLM-T) in AF, with increased CD45 and thrombospondin signaling between CLM-T and other T cell subclusters. qRT-PCR in a mouse AF model confirmed significant gene upregulation in atrial tissue.

**Conclusion:**

This study synthesizes bulk and single-cell transcriptomic analyses to identify genes associated with lactate metabolism as potential biomarkers for AF and to elucidate T cell alterations in AF. These findings offer novel insights into the pathogenesis of AF and suggest potential strategies for its diagnosis.

## 1 Introduction

Atrial fibrillation (AF), the most prevalent sustained cardiac arrhythmia, affects 1%–2% of the global population and significantly contributes to stroke and heart failure-related mortality (Wong et al., 2024; Shi et al., 2022). Despite advances in catheter ablation and anticoagulation therapies, AF recurrence rates remain high, partially due to persistent atrial remodeling and inflammation, underscoring the need to unravel its pathogenesis underpinnings (Winkle et al., 2023).

Beyond electrophysiological remodeling, emerging evidence implicates immune microenvironment dysregulation in AF progression (Scott et al., 2021; Huang et al., 2024; Chen et al., 2024). T lymphocytes, particularly CD4^+^ and CD8^+^ subsets, emerge as key mediators: CD8^+^ T cell senescence marks AF atrial tissue and correlates with recurrence (Li et al., 2024); CD4^+^CD28^null^ T cells predict postoperative AF and heart failure outcomes (Sulzgruber et al., 2017; Sulzgruber et al., 2018; Hammer et al., 2021); Th17/Treg imbalance associated with AF-related inflammation and fibrosis (Wu et al., 2016; He et al., 2018; Chen et al., 2020). These observations collectively suggest that T cell-driven immunity may represent a potential therapeutic target for AF. Meanwhile, lactate has been recognized as an active immunometabolic regulator, modulating T cell function via histone lactylation, HIF-1α stabilization, and GPR81 signaling (Naik et al., 2025; Zhang et al., 2025; Caslin et al., 2021). In cancer, lactate promotes Treg accumulation and PD-1 upregulation (Yasukawa et al., 2025; Wang et al., 2025). However, whether lactate metabolism similarly contributes to T cell alteration in AF remains unknown.

Here, we integrated bulk and single-nucleus transcriptomic data to identify lactate metabolism-related genes using Weighted Gene Co-expression Network Analysis (WGCNA) and machine learning, and to explore their associations with T cell subset dynamics in AF, validated key genes by AF mouse model, thereby providing a basis for future studies.

## 2 Methods

### 2.1 Data acquisition and preprocessing

Bulk RNA-seq datasets from AF and sinus rhythm (SR) patients were retrieved from the GEO database: GSE79768 (discovery cohort, n = 13) and GSE41177 (validation cohort, n = 20) (Platform GPL570) (Tsai et al., 2016; Yeh et al., 2013). Lactate metabolism-related genes (LRGs, n = 387) were curated from the MsigDB (https://www.gsea-msigdb.org/gsea/msig db/) (Castanza et al., 2023).

### 2.2 Differential expression analysis and functional annotation

Differentially expressed genes (DEGs) between AF and SR were identified using limma (v3.62.1) with thresholds: |log2FC| >1 and FDR-adjusted p < 0.05 (Ritchie et al., 2015). Gene Ontology (GO) and KEGG pathway enrichment analyses were performed using clusterProfiler (v4.14.4) with org. Hs.e.g.,.db (v3.20.0) (Wu et al., 2021).

### 2.3 Immune microenvironment profiling

Immune cell infiltration was estimated using CIBERSORT (v1.03), xCell (v1.1.0), and MCPcounter (v1.2.0) (Newman et al., 2015; Aran et al., 2017; Becht et al., 2016). The Wilcoxon test compared cell proportions between groups, and the Spearman correlation assessed gene-immune cell associations.

### 2.4 Weighted gene co-expression network analysis (WGCNA)

A signed network was constructed (WGCNA v1.73) using the top 5,000 most variable genes from GSE79768 (Langfelder and Horvath, 2008). The soft threshold (β = 6) was chosen to satisfy scale-free topology (*R* ^2^ ≥0.9). The AF-associated modules (MEblue and MEturquoise) were selected (MEblue: module-trait correlation r = 0.63, p = 6e-04; MEturquoise: module-trait correlation r = 0.69, p = 1e-04), and its genes which significance and correlation coefficients exceeding 0.5 were intersected with DEGs and LRGs to identify key genes.

### 2.5 Gene set enrichment analysis (GSEA) and transcriptional regulatory network analysis

GSEA was performed using the clusterProfiler package in R to identify functionally enriched pathways based on the GSE79768 dataset. Gene expression correlations were calculated using Spearman correlation, and genes were ranked accordingly. Gene symbols were converted to ENTREZ IDs via org. Hs.e.g.,.db, and redundant entries were removed. The ranked gene list was analyzed against the MSigDB using preranked GSEA, with significance set at an adjusted p-value < 0.05. GSEA enrichment plots were generated to visualize the enrichment score profiles of significantly enriched gene sets, illustrating their distribution across the ranked gene list. Transcription factor (TF) prediction based on hTFtarget (http://bioinfo.life.hust.edu.cn/hTFtarget#!/). TFs that regulate all key genes simultaneously were retained. The regulatory network was visualized in Cytoscape (v3.10.0) (Shannon et al., 2003).

### 2.6 Diagnostic model development and validation

Three machine learning algorithms were compared: Random Forest (RF), Support Vector Machine (SVM), and Generalized Linear Model via Elastic Net Regularization (GLMnet) (Hu and Szymczak, 2023; Yang et al., 2014; Nelder and Wedderburn, 1972). Key genes were selected as features. The GSE79768 dataset was used for the construction of the model. Model performance was evaluated via 10-fold cross-validation repeated 10 times. The optimal model was selected based on: the area under the ROC curve (AUC) and model residuals. Decision Curve Analysis (DCA) and calibration curve analyses were used to evaluate the model. External validation was performed on GSE41177 datasets.

### 2.7 SnRNA-seq analysis

SnRNA-seq data (GSE255612) were processed using Seurat (v5.1.0) (Hao et al., 2023; Hill et al., 2024). Data were normalized via SCTransform. Principal Component Analysis (PCA) identified the top 20 PCs for t-distributed stochastic neighbor embedding (t-SNE) clustering (resolution = 0.5). Cell clusters were annotated using manual curation based on existing literature and gene characteristics (Hill et al., 2024; Shimazu et al., 2016). T cells were subsetted and re-clustered (resolution = 0.2). Metabolic pathway activity was scored by AUCell (v1.24.0) based on HALLMARK and KEGG gene sets. Cell-cell communication analysis employed CellChat (v1.6.1) with default ligand-receptor pairs (Jin et al., 2021).

### 2.8 AF animal model and experimental procedures

A total of 10 C57BL/6 J male mice (8-week-old) were obtained from the Department of laboratory Animals, Central South University. The animal breeding process and experimental procedures adhered to protocols sanctioned by the Central South University Animal Care and Use Committee. All animal studies were reported according to the ARRIVE guidelines (Kilk et al., 2010). Specifically, the study included the following treatment groups: control (saline, n = 5) and AF model (Ach-CaCl_2_; n = 5). Baseline transthoracic echocardiography and electrocardiogram (ECG) recordings were performed on all mice to ensure no pre-existing differences between groups. AF model was established by a daily mixture of acetylcholine (66 μg/kg; Shanghai Macklin Biochemical Co., Ltd., Shanghai, China) and CaCl2 (10 mg/kg; Shanghai Macklin Biochemical Co., Ltd., Shanghai, China) in a total volume of 0.1 mL by tail vein injection (i.v.) for 3 weeks. The control group received daily injections of an equivalent volume of sterile saline (Liu et al., 2021). At the end of the three-week intervention period, a final round of echocardiography and ECG examinations was conducted on all mice before they were euthanized for atrial tissue sampling.

### 2.9 Transthoracic echocardiography and electrocardiogram

During all procedures, mice were lightly anesthetized via inhalation of 1.5%–2.0% isoflurane and placed on a heating pad to maintain body temperature at 37°C. Transthoracic echocardiography was performed using a Vevo F2 imaging system and analyzed with the accompanying Vevo LAB software (FUJIFILM VisualSonics, Toronto, Canada) to assess cardiac structure and function. Key parameters, including left atrial diameter (LAD) and left ventricular ejection fraction (LVEF), were measured from the parasternal long-axis view. All assessments were conducted by a technician blinded to the experimental groups. Subsequently, surface ECGs were recorded and analyzed using a BL-420N biological signal acquisition and processing system (Chengdu TME Technology Co., Ltd., Chengdu, China).

### 2.10 Quantitative real-time PCR (qRT-PCR) analysis

Total RNA was isolated from mouse atrial tissue using the MiniBEST Universal RNA Extraction Kit (Takara Bio, Kyoto, Japan). cDNA was synthesized with the PrimeScript RT reagent Kit with gDNA Eraser (Takara Bio). Quantitative real-time PCR was performed using TB Green Premix Ex Taq II under the following cycling conditions: initial denaturation at 95°C for 30 s, followed by 40 cycles of 95°C for 5 s and 60°C for 30 s, with a final extension at 65°C for 5 s and 95°C for 5 s, in a total reaction volume of 10 µL. Gene expression levels were calculated using the 2^-∆∆Ct^ method and normalized to GAPDH.

The primer sequences used were listed as follows:

GAPDH (forward 5′‐GGAGCGAGATCCCTCCAAAT‐3´; reverse 5′‐GGCTGTTGTCATACTTCTCATGG‐3′).
SLC16A1 (forward 5′‐TGGCTGTCATGTATGGTGGAGGTC‐3´; reverse 5′‐GAAGCTGCAATCAAGCCACAGC‐3′).
MRPL44 (forward 5′‐TTGAAGACGAGTACCCAGACA‐3´; reverse 5′‐GGGCTCCAATAACTGCAAAGAA‐3′).
FLI1 (forward 5′‐GGATGGCAAGGAACTGTGTAA‐3´; reverse 5′‐GGTTGTATAGGCCAGCAG‐3′).
COX16 (forward 5′‐CACAAATCCGGTACGATGCTG‐3´; reverse 5′‐GGAGTTGAGGATCTTCCCAAGG‐3′).
COG3 (forward 5′‐GATGGGAGACCCGACTCGAT‐3´; reverse 5′‐GCAGCGACTGGGATGCTAA‐3′).
CD46 (forward 5′‐GGCCAGATAAGTTTTCCCTTGT‐3´; reverse 5′‐AGGCTTGGTAGGATGAGTAGG‐3′).

### 2.11 Statistical analysis

All analyses were performed in R v4.4.1 and GraphPad Prism 9 software. Continuous variables were compared between groups using the Student’s t-test or the Wilcoxon rank-sum test, as appropriate. Categorical variables were compared using Chi-square test or Fisher’s exact test. Correlation analyses used Spearman’s rank coefficient. P < 0.05 was considered statistically significant.

## 3 Results

### 3.1 Different expression gene and cardiac immune microenvironment characteristics in AF patients

**FIGURE 1:**
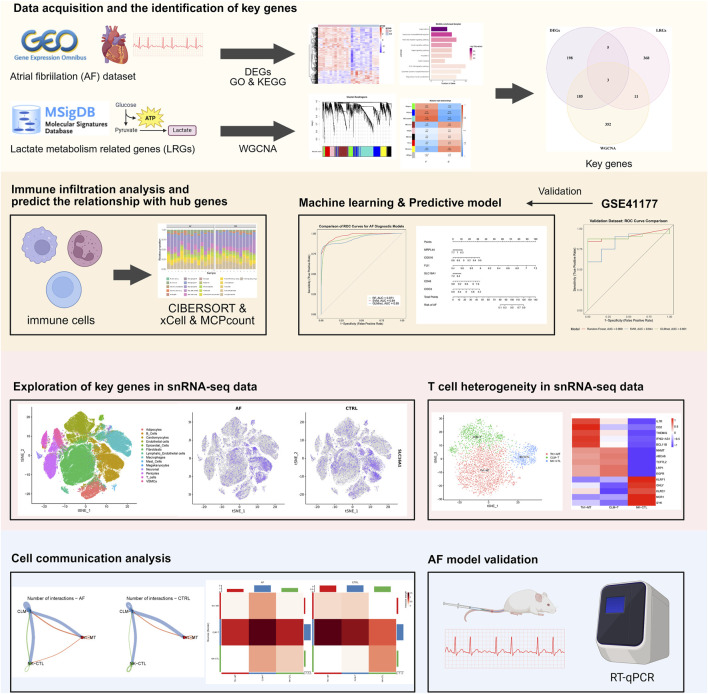
Flowchart of the research workflow.

**FIGURE 2:**
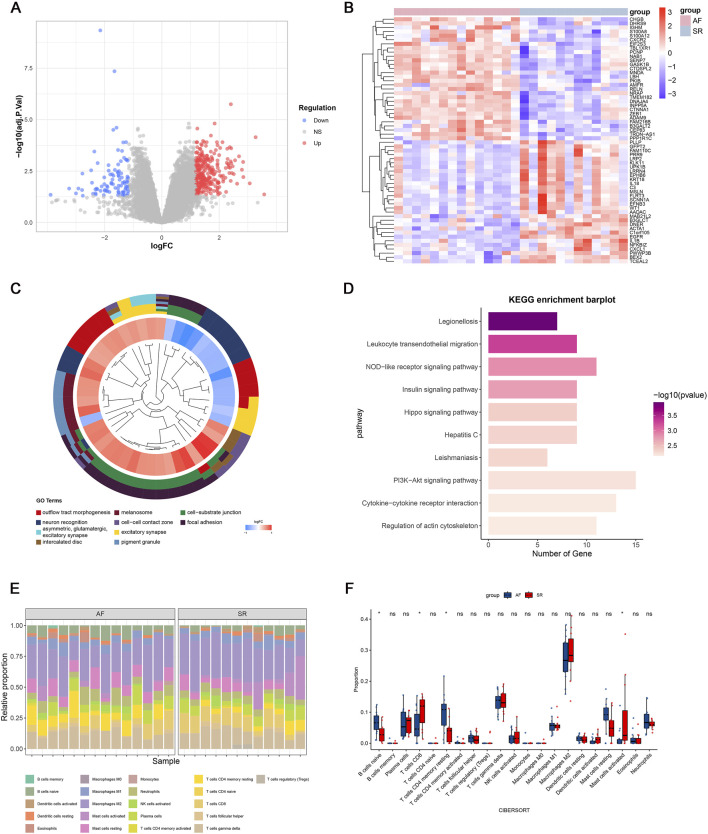
Gene expression profiles associated with atrial fibrillation (AF). **(A)** Volcano plot of differentially expressed genes (DEGs); **(B)** Heatmap of the top 30 upregulated and downregulated genes; **(C)** Top 10 enriched Gene Ontology (GO) terms; **(D)** Top 10 enriched KEGG pathways; **(E)** Immune cell infiltration landscape in GSE79768; **(F)** Differential immune cell infiltration between AF and sinus rhythm (SR) groups (p < 0.05; ns, not significant).

The study workflow is summarized in Figure 1. We identified 391 differentially expressed genes (DEGs), including 300 upregulated and 91 downregulated genes. Hierarchical clustering of the top 30 upregulated and downregulated DEGs distinctly separated atrial fibrillation (AF) and sinus rhythm (SR) samples (Figures 2A,B). GO and KEGG enrichment analyses showed significant enrichment in leukocyte transendothelial migration and cytokine signaling pathways, implicating immune-inflammatory processes in AF (Figures 2C,D). CIBERSORT analysis revealed altered T cell homeostasis in AF, with increased resting memory CD4^+^ T cells and decreased CD8^+^ T cells (Figures 2E,F; p < 0.05). These results suggest that AF is associated with immune microenvironment dysregulation, particularly involving T cell subset alteration.

Recent studies have shown that metabolic reprogramming, particularly lactate metabolism, plays a key role in T cell differentiation and function. Given our observation of abnormal T cell subset proportions in AF patients, we hypothesized that lactate metabolism-related genes may contribute to the immune imbalance associated with AF. Therefore, we further investigated the involvement of lactate metabolism genes in AF by identifying relevant key genes through integrative analysis.

### 3.2 Identification of key genes through WGCNA for predicting AF

**FIGURE 3:**
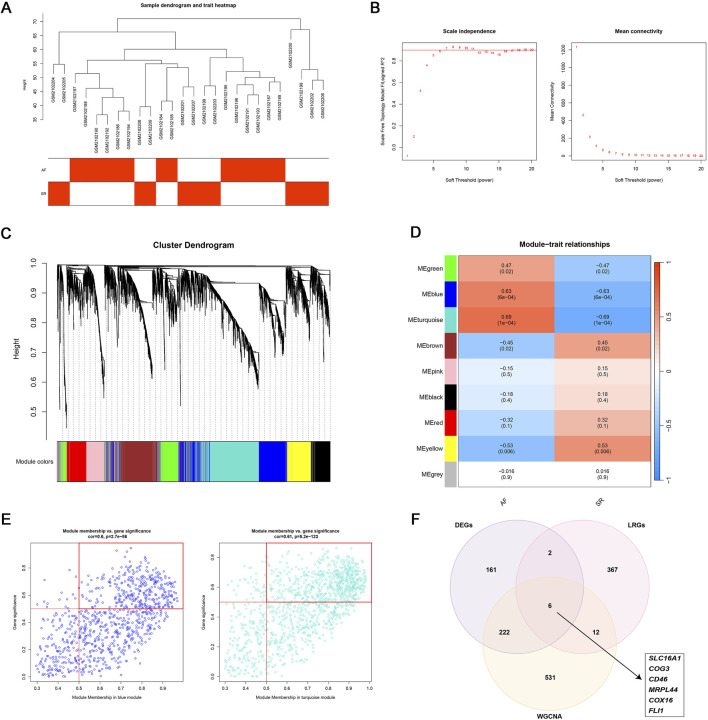
WGCNA analysis. **(A–D)** WGCNA identified two AF-associated modules: The MEblue and MEturquoise modules showing positive correlation with AF; **(E)** High-correlation genes (correlation coefficient >0.5) within MEblue and MEturquoise; **(F)** Six lactate metabolism-related key genes linked to AF.

To identify AF-associated key genes potentially involved in lactate metabolism and immune dysregulation, WGCNA was performed on AF and SR cohorts. A soft threshold (β = 6) achieved scale-free topology (*R* ^2^ ≥0.9, slope = −1.2; Figures 3A,B). The blue (r = 0.63, p = 6 × 10^−4^) and turquoise (r = 0.69, p = 1 × 10^−4^) modules showed strongest AF correlation (Figures 3C,D). Genes from these modules with high module significance (gene significance >0.5 and module membership >0.5) were intersected with the differentially expressed genes (DEGs), resulting in 228 core candidates relevant to AF. To specifically investigate the role of lactate metabolism, we further intersected these 228 genes with a predefined set of 387 lactate metabolism-related genes (LRGs; see Methods). This integrative screening identified six key genes: SLC16A1, MRPL44, FLI1, COX16, COG3, and CD46 (Figures 3E,F).

**FIGURE 4:**
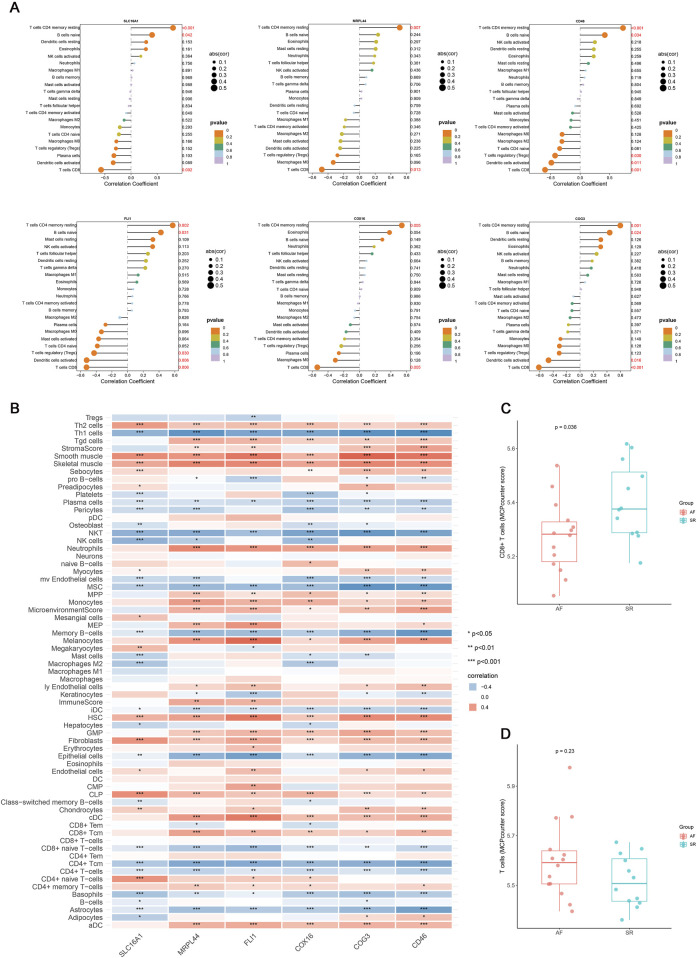
The correlation analysis results of key genes with immune cells. **(A)** Lollipop plot of the correlation between six key genes (SLC16A1, MRPL44, FLI1, COX16, COG3, CD46) in the mRNA expression profile and immune cells identified by CIBERSORT. **(B)** Correlation heatmap of six key genes expression with immune cell infiltration identified by xCell (*p < 0.05, **p < 0.01, ***p < 0.001). **(C)** Boxplot of the difference in CD8^+^ T cells relative abundance between AF and SR groups. **(D)** Boxplot of the difference in T cells relative abundance between AF and SR groups.

**FIGURE 5:**
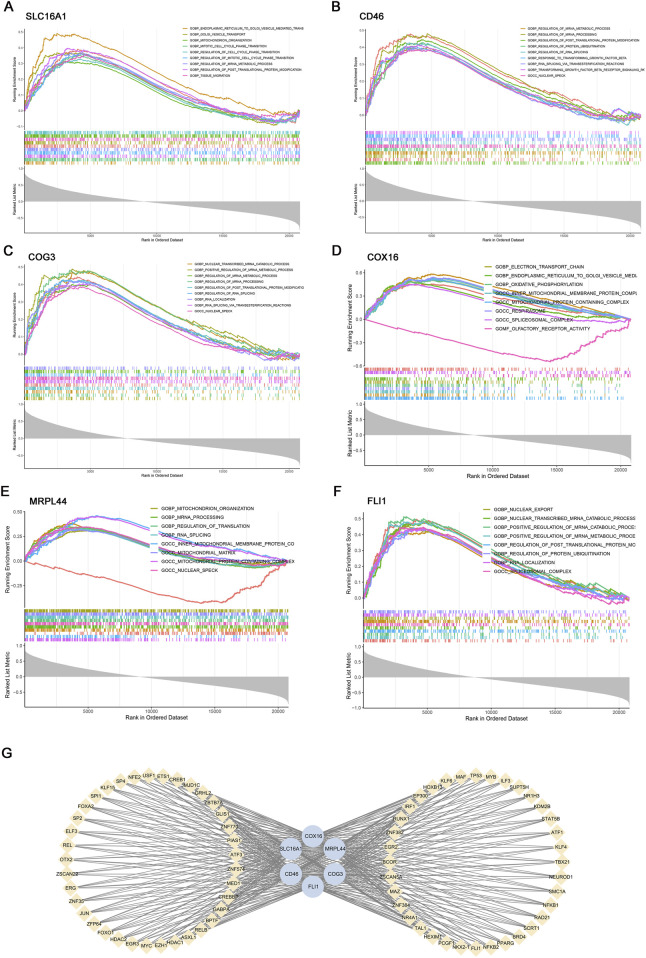
Gene set enrichment analysis (GSEA) and transcription factor (TF) prediction of six key genes. GSEA of key genes: **(A)** SLC16A1, **(B)** CD46, **(C)** COG3, **(D)** COX16, **(E)** MRPL44, **(F)** FLI1. **(G)** Transcriptional regulatory network of key genes predicted by hTFtarget and visualized in Cytoscape.

Multiple immune deconvolution methods (CIBERSORT, xCell, and MCPcounter) consistently demonstrated the T cell subset alteration in AF and all six key genes were positively associated with resting memory CD4^+^ T cells and negatively with CD8^+^ T cells (p value < 0.05, r >0.4, Figures 4A–D). To elucidate their potential functions, we performed pathway enrichment and transcriptional network analyses (Figure 5). These findings suggest that the identified lactate metabolism genes may contribute to immune microenvironment dysregulation in AF.

### 3.3 Diagnostic model development and validation

**FIGURE 6:**
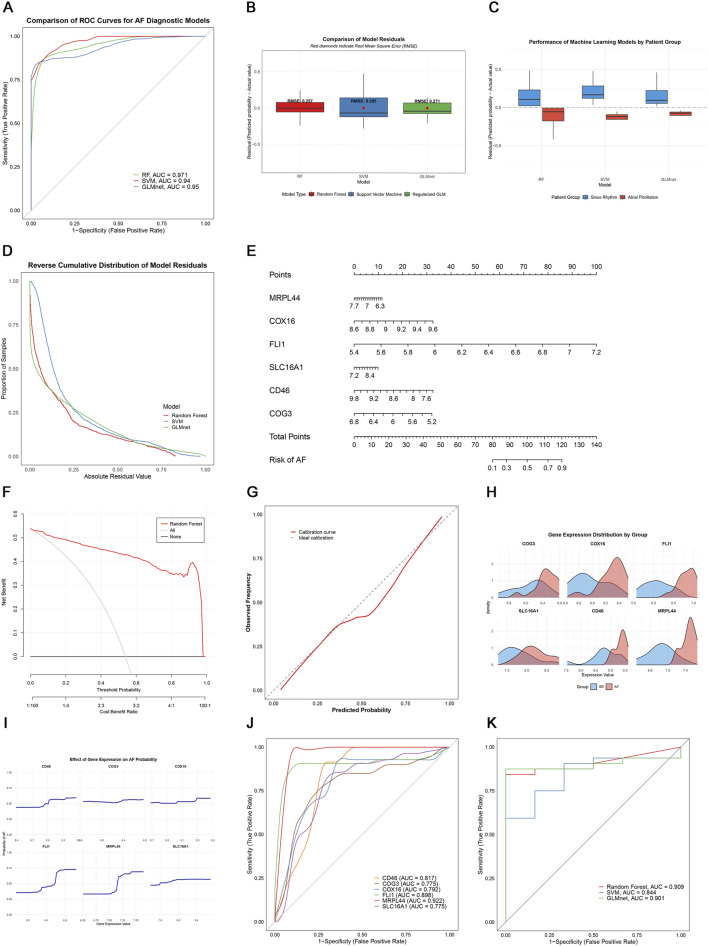
Machine learning-based AF diagnosis. **(A)** ROC curves of three models; **(B,C)** Residual comparisons between RF, SVM, and GLMnet; **(D)** Reverse cumulative residual distributions; **(E)** Nomogram for AF risk prediction; **(F)** Decision curve analysis showing the net benefit of the gene model (red) versus treat-all (gray) and treat-none (black) strategies; **(G)** Calibration curve; **(H)** Key gene expression distribution; **(I)** Gene effect on AF probability; **(J)** Individual gene ROC curves; **(K)** External validation in GSE41177.

To assess the diagnostic potential of lactate metabolism-related genes (LRGs), we compared three machine learning models: random forest (RF), support vector machine (SVM), and GLMnet. Among these, the RF model exhibited the best performance, with the lowest residual errors and the highest area under the curve (AUC = 0.971), outperforming both SVM (AUC = 0.94) and GLMnet (AUC = 0.95) (Figures 6A–D). Decision curve and calibration curve analyses further supported the superior net benefit and prediction accuracy of the RF model (Figures 6F,G).

We constructed an RF-based nomogram that integrated all six key genes (SLC16A1, MRPL44, FLI1, COX16, COG3, and CD46), enabling individualized risk prediction for AF (Figure 6E). Receiver operating characteristic (ROC) analysis of the six key genes showed that CD46, FLI1, and MRPL44 each achieved AUC values above 0.8, while SLC16A1 had the lowest AUC at 0.775, indicating robust classification performance (Figure 6J).

External validation using the independent GSE41177 dataset further demonstrated the generalizability of the models, with the RF model achieving an AUC of 0.909, GLMnet an AUC of 0.901, and SVM an AUC of 0.844 (Figure 6K). Together, these results suggest that LRGs are promising biomarkers for AF detection, and that the RF-based model offers a clinically useful tool for risk stratification.

### 3.4 Single-nucleus RNA sequencing visualization of key gene expression

**FIGURE 7:**
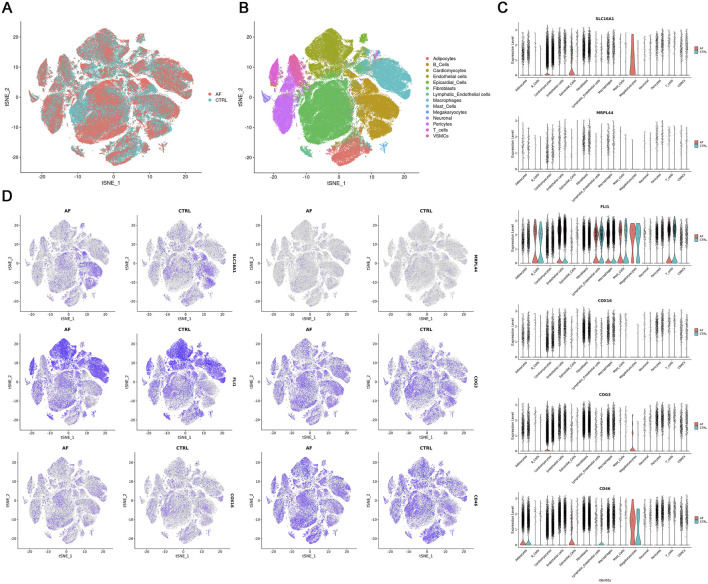
Single-nucleus RNA-seq analysis of AF and CTRL hearts (GSE255612). **(A,B)** t-SNE visualization of 14 annotated cell clusters; **(C)** Violin plots of key gene expression across cell types; **(D)** t-SNE plots showing key gene expression patterns.

To visualize the cell-type specific expression patterns of lactate metabolism-related key genes, we analyzed snRNA-seq data from AF and control hearts (GSE255612). After quality control and normalization, unsupervised clustering identified 14 distinct cell types, including cardiomyocytes, endothelial cells, and immune subsets (T cells, B cells, macrophages), based on canonical marker expression (Figures 7A,B). The six key genes exhibited distinct expression patterns across cell types: COG3, CD46, COX16, and MRPL44 were broadly expressed, SLC16A1 was mainly enriched in cardiomyocytes, and FLI1 was predominantly localized to endothelial cells and immune populations (Figures 7C,D). This cell type-resolved expression indicates their potential involvement in cell-specific pathways in AF pathophysiology.

### 3.5 T cell heterogeneity and metabolic remodeling in AF

**FIGURE 8:**
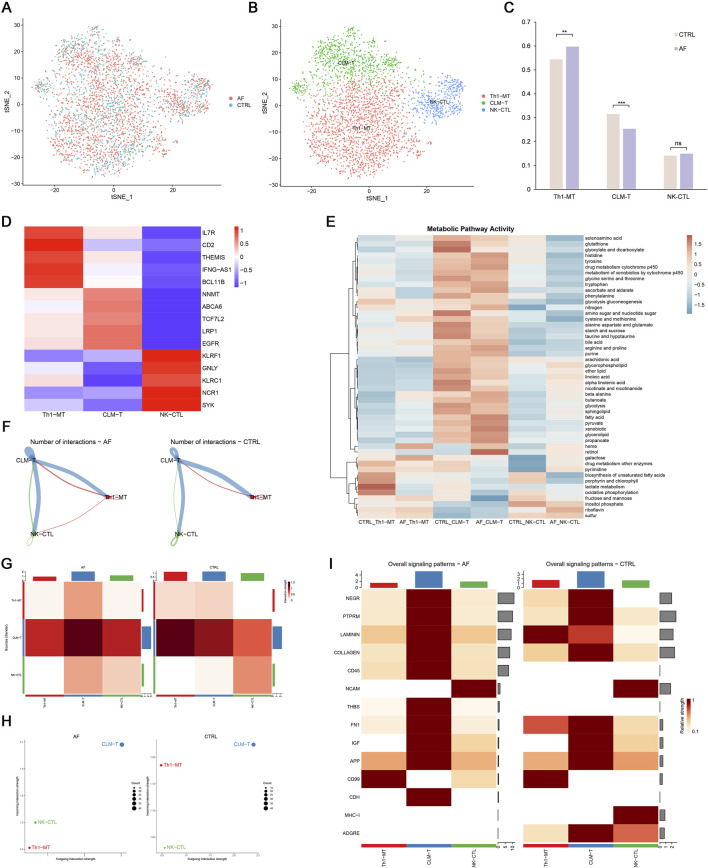
T cell heterogeneity and cellular communication in AF. **(A,B)** t-SNE plots of 3 T cell subsets; **(C)** Proportion differences between AF and control (**p < 0.01, **p < 0.001, ns = not significant); **(D)** Heatmap of T cell subset markers; **(E)** Metabolic pathway activity (AUCell scores); **(F,G)** Interaction number and strength; **(H)** Incoming/outgoing interaction strength; **(I)** Differential signaling pathways between groups.

To further dissect T cell heterogeneity in AF, we performed unbiased clustering of T cells, identifying three distinct subsets: (1) Th1-polarized memory T cells (Th1-MT, marked by IL7R, CD2, THEMIS, IFNG-AS1, BCL11B), (2) cardiac lipid-adapted memory T cells (CLM-T, characterized by NNMT, ABCA6, TCF7L2, LRP1, EGFR), and (3) NK-like cytotoxic T cells (NK-CTL, expressing KLRF1, GNLY, KLRC1, NCR1, SYK) (Figures 8A–D). Functionally, Th1-MT are associated with pro-inflammatory responses, CLM-T with metabolic adaptation, and NK-CTL with cytotoxicity.

Comparative analysis revealed that AF hearts exhibited a significantly increased proportion of Th1-MT and a decreased frequency of CLM-T compared to controls (p < 0.05; Figure 8C). Using AUCell-based pathway scoring, we found that CLM-T displayed the highest overall metabolic activity, with significant enrichment in glycolysis and fatty acid metabolism pathways. Notably, Th1-MT showed significantly elevated lactate metabolism activity compared to the other subsets (p < 0.05; Figure 8E), indicating subset-specific metabolic adaptations in the context of AF.

### 3.6 Altered intercellular communication in AF

Cell-cell communication analysis demonstrated enhanced interaction frequency and signal strength in AF T cells, particularly involving CLM-T as a central signaling node (Figures 8F–H). Pathway-specific changes included upregulation of CD45 and THBS (thrombospondin) pathways (associated with immune activation and adhesion) and downregulation of LAMININ and MHC-I pathways (linked to matrix interaction and antigen presentation) in AF (Figure 8I). These shifts suggest a reconfiguration of T cell communication networks in AF, potentially contributing to immune microenvironment dysregulation.

### 3.7 Experimental validation of the six key genes in an AF mouse model

**FIGURE 9:**
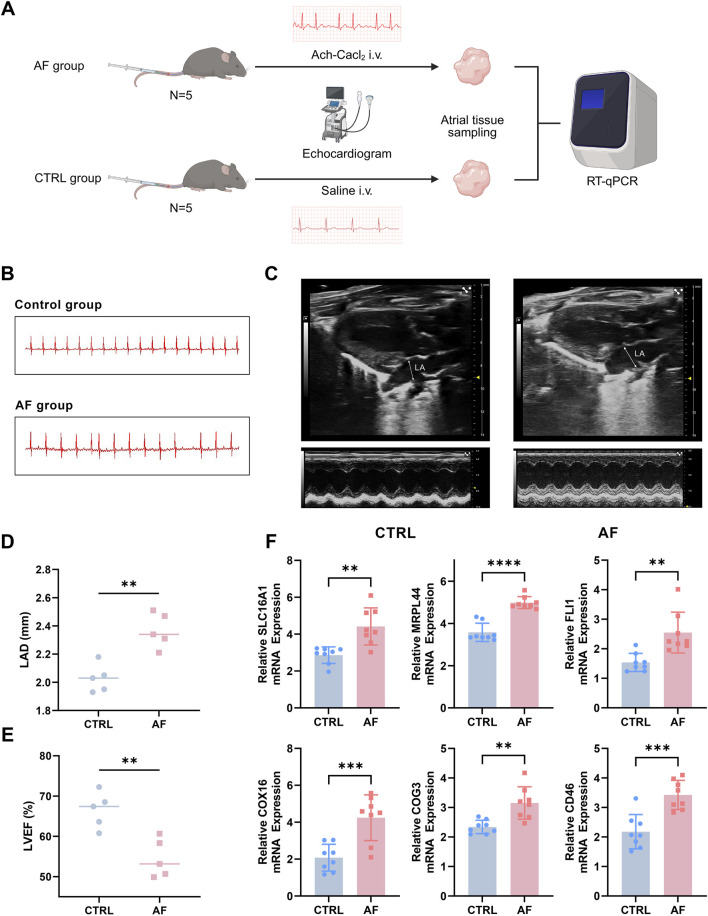
Experimental validation of key genes in an AF mouse model. **(A)** Flow chart of AF mouse model establishment. **(B)** Representative ECG traces showing loss of P waves and irregular rhythm in AF mice. **(C)** Typical echocardiography of the heart in AF model group and control (CTRL) group. **(D,E)** Increased left atrial diameter (LAD) and reduced left ventricular ejection fraction (LVEF) in AF mice versus controls, assessed by echocardiography. **(F)** qRT-PCR analysis reveals significant upregulation of six key genes (SLC16A1, MRPL44, FLI1, COX16, COG3, CD46) in atrial tissue of AF mice compared to controls (**P < 0.01, ***P < 0.001, ****P < 0.0001).

Prior to the intervention, baseline echocardiography and ECG parameters showed no significant differences between the two groups (p > 0.05) (Supplementary Figure S1; Supplementary Table S1). To experimentally validate the expression levels of the six key genes identified in our analysis, we established an AF mouse model as described in the Methods section (Figure 9A). ECG analysis confirmed successful induction of AF in the model group, as evidenced by the disappearance of P waves and the presence of an irregularly irregular rhythm, which are typical hallmarks of AF (Figure 9B). In addition, echocardiography revealed significantly increased LAD and reduced LVEF in the AF group compared to controls (CTRL), further supporting the successful establishment of the AF phenotype (Figures 9C–E).

Subsequently, the mRNA expression levels of the six key genes (SLC16A1, MRPL44, FLI1, COX16, COG3, and CD46) were measured using qRT-PCR. The results demonstrated that all six genes were significantly upregulated in the atria of AF mice compared to controls (Figure 9F; p < 0.05 for all genes, Student’s t-test). These experimental findings corroborate our bioinformatics analyses and highlight the potential contribution of these genes in AF pathophysiology.

## 4 Discussion

Despite advances in pharmacotherapy and catheter ablation, atrial fibrillation (AF) management remains hampered by variable treatment responses and high recurrence rates (Arcoraci et al., 2025; Parks et al., 2024). While electrical and structural remodeling are important in the pathogenesis of AF, increasing evidence suggests that metabolic-immune interactions may also contribute to disease development, supporting the need for new biomarker identification (Tian et al., 2023; Zhao et al., 2023).

In this study, we used an integrated transcriptomic analysis approach to identify six lactate metabolism-related genes (SLC16A1, MRPL44, FLI1, COX16, COG3, and CD46) that were associated with CD4^+^/CD8^+^ T cell imbalance of AF (Floyd et al., 2023). The diagnostic model constructed from these genes demonstrated favorable performance in public datasets. Furthermore, in an AF mouse model that recapitulated key features of the human disease—including characteristic ECG changes, increased left atrial diameter, and reduced left ventricular function—qRT-PCR analysis indicated that all six genes were significantly upregulated in atrial tissue of AF mice compared to controls. These results provide preliminary support for the relevance of these genes in AF, though the associations observed here require further mechanistic investigation. It should also be noted that mouse models, while informative, may not fully reflect the complexity of human AF.

Our single-cell analysis showed that the CLM-T subset, characterized by enrichment in glycolytic and fatty acid metabolism genes, was reduced in AF samples. Metabolic features partially overlapping with CLM-T have been reported in exhausted T cells in tumor microenvironments, hinting at potential parallels in adaptation mechanisms under stress (Chen et al., 2025). We also observed higher lactate metabolism pathway activity in control Th1-MT cells compared to other T cell subsets, consistent with previous reports implicating glycolysis in Th1 differentiation (Salles et al., 2023; Zhang et al., 2023). However, the biological significance of the observed changes in CLM-T and Th1-MT metabolic activity in AF remains to be established, and further functional studies are needed to clarify these findings (Lee et al., 2025).

CellChat analysis predicted enhanced CD45 and THBS signaling pathways between CLM-T and other T cell subsets in AF, suggesting possible alterations in intercellular communication. The involvement of the CD45 pathway is noteworthy due to its known role in T cell receptor signaling and immune regulation (Samarakoon et al., 2016). Likewise, THBS1-mediated interactions may influence the pro-fibrotic microenvironment via effects on TGF-β activation and T cell function (Kresoja et al., 2021; Wei et al., 2024; Li et al., 2023). Similar dysregulation of these pathways in other immune-mediated diseases, such as rheumatoid arthritis, suggests the possibility of shared mechanisms (Rico et al., 2008; Jiang et al., 2025; Rider et al., 2013). However, further studies are needed to determine their functional relevance in AF.

Alterations in the lactate metabolism-related gene profile and T cell subpopulation observed here may be comparable to findings in other conditions, such as cancer and heart failure, where metabolic stress is thought to drive immune dysregulation. Bidirectional crosstalk involving lactate-modulated histone lactylation and cytokine-driven metabolic shifts has been described (Naik et al., 2025; Hu et al., 2024; Kim et al., 2025). Our results suggest that such metabolic–immune interactions may also occur in AF, but more work is needed to clarify these mechanisms and their clinical implications.

## 5 Limitations

This study has several limitations. Although we validated gene expression changes in an AF mouse model, we did not directly assess the corresponding immune cell alterations, such as T cell infiltration, in the atrial tissue. Direct functional studies are needed to elucidate the mechanistic roles of these genes in AF. The sample size was relatively small, which may affect the robustness of subgroup analyses. In addition, we did not evaluate the association of these biomarkers with clinical outcomes or treatment response in human patients. Future studies should include comprehensive functional validation using both *in vitro* and *in vivo* models, larger patient cohorts, and mechanistic exploration of the metabolic-immune axis in AF. Furthermore, it is important to acknowledge that while this drug-induced model was effective for validating our transcriptomic findings, a pharmacologically-driven arrhythmia may not fully recapitulate the complex and progressive structural and immune remodeling characteristic of chronic human AF.

## 6 Conclusion

In conclusion, our comprehensive transcriptomic analysis indicates that genes associated with lactate metabolism may serve as potential biomarkers for AF, representing promising targets for further exploration in diagnostic and therapeutic contexts. Additionally, T cells exhibited alterations in metabolic transcriptomics in the context of AF. These findings enhance our understanding of AF pathogenesis; however, further validation and functional studies are necessary before considering clinical applications.

## Data Availability

The raw data supporting the conclusions of this article will be made available by the authors, without undue reservation.
