# Supplementary material for: Integrated single-cell and bulk transcriptome analysis reveal lactate metabolism-related signature and T cell alteration in atrial fibrillation
Source: Front Cell Dev Biol. 2025 Aug 6;13:1644702. doi: 10.3389/fcell.2025.1644702 (PMC12365808; doi:10.3389/fcell.2025.1644702)
Supplement: Supplementary file 1 [file Table1.docx]

**Table S1. Baseline echocardiography data for individual mice in the control and AF groups.**

| **Parameter** | **CTRL Group (n=5)** | **AF Group (n=5)** | **p-value** |
| --- | --- | --- | --- |
| **LAD (mm)** |  |  | 0.54¹ |
| Mouse 1 | 2.16 | 2.22 |  |
| Mouse 2 | 2.05 | 2.08 |  |
| Mouse 3 | 2.21 | 2.17 |  |
| Mouse 4 | 2.08 | 2.03 |  |
| Mouse 5 | 2.14 | 1.97 |  |
| **Mean ± SEM** | **2.13 ± 0.03** | **2.09 ± 0.04** |  |
| **LVEF (%)** |  |  | 0.87¹ |
| Mouse 1 | 64.72 | 68.44 |  |
| Mouse 2 | 70.65 | 62.15 |  |
| Mouse 3 | 67.13 | 70.82 |  |
| Mouse 4 | 69.21 | 65.77 |  |
| Mouse 5 | 60.93 | 63.56 |  |
| **Mean ± SEM** | **66.53 ± 1.64** | **66.15 ± 1.39** |  |

¹ P-values were calculated using Student’s t-test. Data are presented as individual values and as mean ± SEM. LAD, left atrial diameter; LVEF, left ventricular ejection fraction.
