# Supplementary figures and images for: Integrated single-cell and bulk transcriptome analysis reveal lactate metabolism-related signature and T cell alteration in atrial fibrillation
Source: Front Cell Dev Biol. 2025 Aug 6;13:1644702. doi: 10.3389/fcell.2025.1644702 (PMC12365808; doi:10.3389/fcell.2025.1644702)

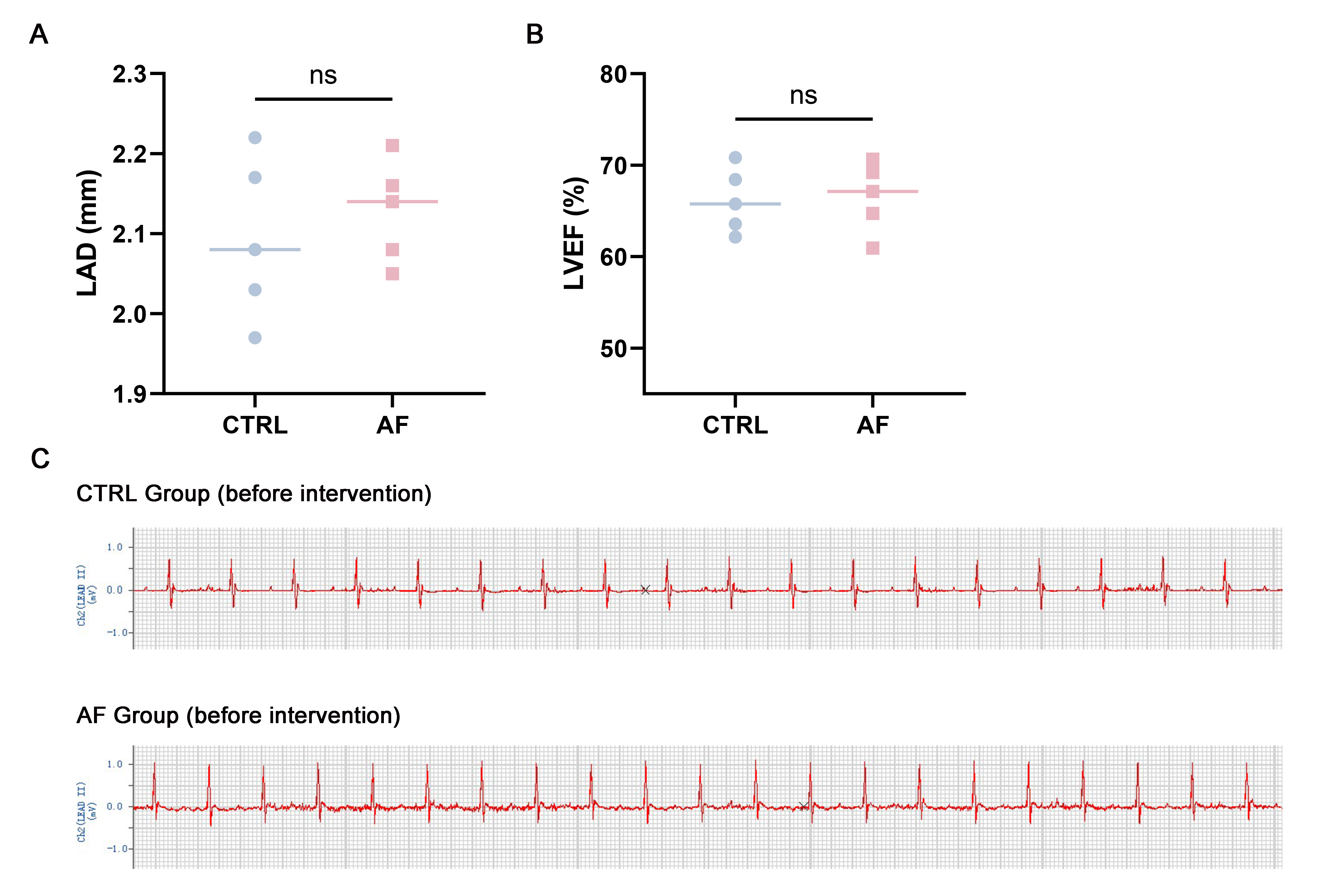

Supplement: Supplementary file 2 [file Image1.jpeg]
